# Assessment of the underlying systems involved in standing balance: the additional value of electromyography in system identification and parameter estimation

**DOI:** 10.1186/s12984-017-0299-x

**Published:** 2017-09-15

**Authors:** J. H. Pasma, J. van Kordelaar, D. de Kam, V. Weerdesteyn, A. C. Schouten, H. van der Kooij

**Affiliations:** 1 Department of Biomechanical Engineering, Delft University of Technology, Mekelweg 2, 2628 CD Delft, The Netherlands; 2 Department of Biomechanical Engineering, Institute for Biomedical Technology and Technical Medicine (MIRA), University of Twente, Enschede, The Netherlands; 3 Department of Rehabilitation, Donders Institute for Brain, Cognition and Behaviour, Radboud University Medical Center, Nijmegen, The Netherlands; 4 Sint Maartenskliniek Research, Nijmegen, The Netherlands

**Keywords:** Posture, Human balance control, Modelling, Muscle activation, Activation dynamics

## Abstract

**Background:**

Closed loop system identification (CLSIT) is a method to disentangle the contribution of underlying systems in standing balance. We investigated whether taking into account lower leg muscle activation in CLSIT could improve the reliability and accuracy of estimated parameters identifying the underlying systems.

**Methods:**

Standing balance behaviour of 20 healthy young participants was measured using continuous rotations of the support surface (SS). The dynamic balance behaviour obtained with CLSIT was expressed by sensitivity functions of the ankle torque, body sway and muscle activation of the lower legs to the SS rotation. Balance control models, 1) without activation dynamics, 2) with activation dynamics and 3) with activation dynamics and acceleration feedback, were fitted on the data of all possible combinations of the 3 sensitivity functions. The reliability of the estimated model parameters was represented by the mean relative standard errors of the mean (mSEM) of the estimated parameters, expressed for the basic parameters, the activation dynamics parameters and the acceleration feedback parameter. To investigate the accuracy, a model validation study was performed using simulated data obtained with a comprehensive balance control model. The accuracy of the estimated model parameters was described by the mean relative difference (mDIFF) between the estimated parameters and original parameters.

**Results:**

The experimental data showed a low mSEM of the basic parameters, activation dynamics parameters and acceleration feedback parameter by adding muscle activation in combination with activation dynamics and acceleration feedback to the fitted model. From the simulated data, the mDIFF of the basic parameters varied from 22.2–22.4% when estimated using the torque and body sway sensitivity functions. Adding the activation dynamics, acceleration feedback and muscle activation improved mDIFF to 13.1–15.1%.

**Conclusions:**

Adding the muscle activation in combination with the activation dynamics and acceleration feedback to CLSIT improves the accuracy and reliability of the estimated parameters and gives the possibility to separate the neural time delay, electromechanical delay and the intrinsic and reflexive dynamics. To diagnose impaired balance more specifically, it is recommended to add electromyography (EMG) to body sway (with or without torque) measurements in the assessment of the underlying systems.

**Electronic supplementary material:**

The online version of this article (10.1186/s12984-017-0299-x) contains supplementary material, which is available to authorized users.

## Background

Impaired balance is a common complaint in elderly and patients with specific diseases like vestibular disorders, stroke or Parkinson’s disease [1–6]. To maintain standing balance, several underlying systems interact, such as the nervous system, sensory systems and motor system. With age, specific diseases and medication use, these systems deteriorate and compensate for each other’s deteriorations [7–10], which makes it difficult to detect the underlying cause of impaired balance. To diagnose and intervene impaired balance with targeted interventions it is important to detect the underlying cause of impaired balance. Closed loop system identification (CLSIT) combined with perturbations is a method to distinguish the contribution of underlying systems in standing balance by taken into account the interrelation between the underlying systems and to describe the underlying systems with physiologically meaningful parameters. This gives the possibility to identify the underlying changes in standing balance and therefore to diagnose impaired balance more specifically [11, 12].

Several studies used CLSIT to assess standing balance in a variety of patient groups, such as elderly [13–16], vestibular loss patients [17], Parkinson’s disease patients [18–20] and stroke patients [21], by describing underlying changes in standing balance with physiologically meaningful parameters. To ‘open’ the closed loop, sensory and/or mechanical perturbations were applied to disentangle cause and effect and to assess the contribution of the sensory systems (i.e. proprioception, vision and vestibular system), sensory reweighting [13, 17], the dynamics of the human body [22, 23] or the (inter)limb stabilization [14, 18–21]. All these studies used measurements of the kinetics and/or kinematics by motion capture systems and force plates to estimate the sensitivity functions to mechanical and/or sensory perturbations, the control dynamics and/or the human body dynamics [24]. Only two of these studies [22, 23] used muscle activation as outcome measure. They used muscle activation in combination with sensory perturbations to identify the human body dynamics including activation dynamics (i.e. the mapping from muscle activation to body segment angles). However, adding muscle activation to CLSIT in combination with sensory perturbations could result in more insight in the underlying mechanisms involved in standing balance, as explained below.

Adding muscle activation to CLSIT makes it possible to separate the neural time delay of the neural pathways, i.e. the transmission and synaptic delay, from the time delay due to the activation dynamics, i.e. the electromechanical delay. Previous studies showed that the lumped time delay in standing balance, consisting of both the neural time delay and the electromechanical delay, increases with age and diseases [13, 15–17]. However, it is unclear whether this is due to changes in the activation dynamics or to changes in the time delay of the other neural pathways. Furthermore, including muscle activation in CLSIT will give more and better insight in the contribution of the intrinsic (i.e. passive) and reflexive (i.e. active) dynamics in standing balance [25], as the muscle activation only represents the contribution of the reflexive dynamics and does not include the contribution of the intrinsic dynamics. Previous studies identifying other human motion control systems, like stabilization of the trunk and of the ankle, wrist and shoulder joint, already used muscle activation and showed the possibility to distinguish the contribution of the intrinsic and reflexive dynamics in the stabilization of joints [26–29].

Adding the muscle activation to CLSIT requires changes to the balance control model, which is used for parameter estimation to describe the underlying systems with physiologically meaningful parameters. A commonly used balance control model is the independent channel (IC) model [17, 30]. This model consists of sensory pathways for each sensory system, the intrinsic and reflexive dynamics, modelled by a PD controller, and an inverted pendulum. To separate the contribution of the electromechanical delay and the neural time delay, the activation dynamics has to be added to the model, which represent the mapping from muscle activation to corrective torques. Furthermore, acceleration feedback might be required in the balance control model to describe the muscle activation response accurately, as previous studies showed that the response of the muscle activation is explained by position, velocity and acceleration feedback [31, 32].

In this study we investigated the additional value of adding muscle activation of the lower legs measured with electromyography (EMG) to the assessment of the underlying systems in standing balance, especially in the assessment of the intrinsic and reflexive contributions and the assessment of the activation dynamics. Furthermore, we investigated whether it is needed to measure both ankle torque and body sway to estimate reliable and accurate parameters, which will indicate whether the experimental set up can be simplified or not. Experimental data of healthy participants were used to investigate the additional value of EMG in identifying the underlying control mechanisms represented by model parameters. To check the accuracy of the estimated parameters, a model validation study was performed.

## Methods

### Participants

Twenty healthy young participants (10 men, age 23.6 years (SD 2.9 years)) participated in the study. The participants gave written informed consent prior to participation. The protocol was approved by the medical ethics committee of The Medical Spectrum, Enschede, the Netherlands and was in accordance with the Declaration of Helsinki.

### Apparatus and recording

**Fig. 1:**
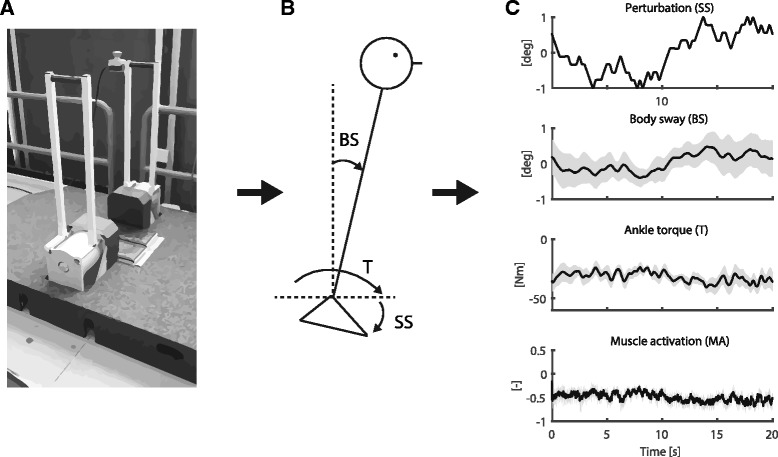
Experimental set up. **a** The Bilateral Ankle Perturbator (BAP) was used to apply the support surface (SS) rotations. **b** The actual SS rotation, body sway (BS) and ankle torque (T) were measured in combination with the muscle activation (MA). **c** A typical example of human reactions on the SS rotation. Time series are presented by mean and standard deviation averaged over data blocks

Figure 1 shows the experimental set up of this study. A Bilateral Ankle Perturbator (BAP) (Forcelink B.V., Culemborg, the Netherlands) was used to disturb the proprioceptive information of both ankles by applying support surface (SS) rotations around the ankle axis (Fig. 1a) [33]. The actual angles of rotation and the applied torques to the left and right SS of the BAP were measured with a sample frequency of 1000 Hz and were stored for further analysis (Fig. 1b).

The body kinematics of the lower and upper body were measured in anterior-posterior direction using two draw-wire potentiometers (Sentech SP2, Celesco, Chatsworth, CA, United States) by connecting them to the participant’s trunk and hip. Together with the motor angles and motor torques, the body dynamics were measured using a Matlab interface with a sample frequency of 1000 Hz and were stored for further analysis (Fig. 1b).

Muscle activity of three lower leg muscles on each side (i.e. the *M. gastrocnemius* medialis, *M. soleus* and *M. tibialis* anterior of both legs) was measured with a wireless EMG system (Cometa Systems, Bareggio, Italy) using pairs of self-adhesive Ag-AgCl surface electrodes, which were placed approximately 2 cm apart and longitudinally on the muscle belly, according to the Seniam guidelines [34]. Together with the motor angles, motor torques and the body dynamics, the EMG was synchronously recorded using Vicon software (Vicon Motion Systems, Oxford, UK) with a sample frequency of 2000 Hz, subsequently down sampled to 1000 Hz and stored for further analysis.

### Procedure

During all experiments the participants stood on the BAP with stocking feet. The participants were instructed to stand with their arms in front of the chest and to keep both feet on the support surface. The support surface rotated following a continuous perturbation signal with an increasing perturbation amplitude over the trials, 0.5, 1, 2, 4 and 8 degrees peak-to-peak, both with eyes open and eyes closed, resulting in 10 trials. Each trial lasted 2 min in which the perturbation signal was 6 times repeated. Before each trial the participant was given sufficient time to get accustomed to the perturbation. The participants wore a safety harness to prevent falling, which did not constrain normal body sway and did not provide support or orientation information.

### Perturbation signal

A pseudorandom ternary sequence (PRTS) of numbers was designed and used as SS angular velocity. Integration of the velocity signal provided an unpredictable perturbation signal of the SS rotation with a wide spectral bandwidth (Fig. 1c) [35]. An 80 state PRTS signal with a time increment of 0.25 s was generated, resulting in a signal with a period time of 20 s. The trial consisted of 6 complete cycles of the perturbation signal resulting in a trial of 2 min.

### Preprocessing

Data analysis was performed with Matlab (The Mathworks, Natick, MA, United States). Leg and hip angles were calculated from potentiometer data resulting in the segment angle of the leg relative to the vertical and the joint angle of the trunk relative to the leg. The body sway was represented by the angle of the Center of Mass (CoM) relative to the vertical, which was calculated using the leg and hip angles and body geometry of individual segments [36].

The data of the motor angles were used to measure the real SS rotation. The ankle torques were obtained from the recorded motor torques.

The EMG signals were combined according to the method described by Kiemel et al. (2008) to obtain one summed EMG signal representing the muscle activation, in which the EMG signals were weighted to optimize the coherence between the perturbation and EMG signal [22]. From each time series of the 6 lower leg muscles the mean was subtracted. The time series were normalized by dividing by their standard deviation computed from all trials for the given subject. Subsequently, each time series was high pass filtered with a bidirectional first-order Butterworth filter with a cut-off frequency of 16 Hz and rectified. To achieve one summed weighted EMG signal for both legs, each time series was multiplied with a weighting factor and summed. The weighting factors were obtained with an optimization function (Matlab function: fmincon) in order to optimize the average coherence between the SS rotation and the summed weighted EMG signal. Average coherence was calculated by averaging the complex coherence (γ_SS,EMG_) across 10 conditions and averaging the squared norm of this coherence (|γ_SS,EMG_|^2^) across the frequencies in the perturbation signal. The complex coherence was calculated using eq. 1

1 $$ {\gamma}_{SS, EMG}(f)={\varPhi}_{SS, EMG}(f){\left[\sqrt{\varPhi_{SS, SS}(f){\varPhi}_{EMG, EMG}(f)}\right]}^{-1} $$

In which Φ_*SS,EMG*_ represents the Cross Spectral Density (CSD) of the SS rotation and the EMG of a specific muscle, Φ_*SS,SS*_ the Power Spectral Density (PSD) of the SS rotation, and Φ_*EMG,EMG*_ the PSD of the EMG of a specific muscle.

The weighting factors were constraint to be positive for dorsal flexors (i.e. left and right *M. tibialis* anterior) and negative for plantar flexors (i.e. left and right *M*. *soleus* and *M*.* gastrocnemius* medialis). In addition, the sum of the absolute weighting factors was constraint to be unity.

The time series of the body sway, SS rotation, ankle torque and muscle activation were segmented into six data blocks of 20 s (i.e. the length of the perturbation signal). Figure 1c shows a typical example of the mean and standard deviation of the SS rotation, body sway, ankle torque and muscle activation.

### Data analysis

**Fig. 2:**
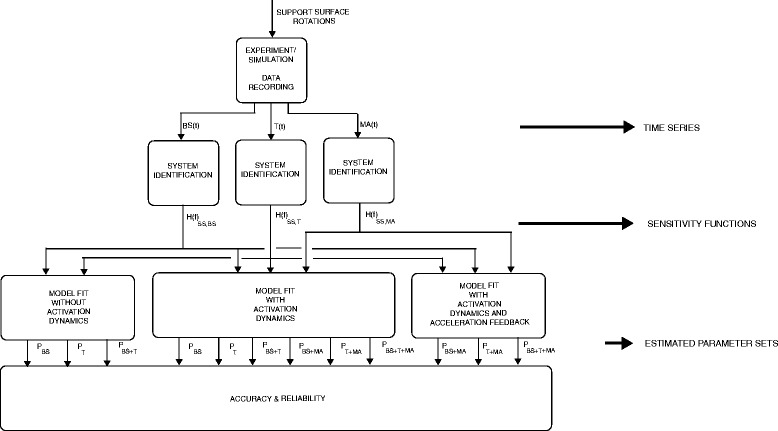
Flowchart of the study set up. The support surface rotations were used in the experiments and simulation to perturb the proprioceptive information, resulting in 3 time series, the body sway (BS(t)), the torque (T(t)) and the muscle activation (MA(t)). Each time series was used to estimate the sensitivity functions resulting in 3 sensitivity functions, sensitivity to support surface rotation of the body sway (H(f)_SS,BS_), of the torque (H(f)_SS,T_) and of the muscle activation (H(f)_SS_,_MA_). All combinations of the sensitivity functions were used in model fits to obtain estimated parameters. Three models were fitted, 1) without activation dynamics, 2) with activation dynamics and 3) with activation dynamics and acceleration feedback. The resulting 12 parameter sets were used to investigate the accuracy and the reliability

Figure 2 shows the flowchart of the data analysis to identify the mechanisms underlying balance behaviour and to investigate the additional value of the muscle activation in the assessment of the underlying systems in standing balance. First, the data obtained from the human experiment were transformed to sensitivity functions using CLSIT. Next, model parameters describing the underlying systems were estimated by fitting a model on the sensitivity functions, in which three models were used, 1) without activation dynamics, 2) with activation dynamics and 3) with activation dynamics and acceleration feedback. The first model was fitted on the sensitivity functions of the body sway, ankle torque or a combination, resulting in 3 parameter sets. The second model was fitted on all possible combinations of sensitivity functions (i.e. muscle activation, body sway and ankle torque), resulting in 6 parameter sets. The third model was fitted on all combination of sensitivity functions including at least the muscle activation, resulting in 3 parameter sets. The parameter sets were used to find the combination of model and sensitivity functions with the most reliable estimated parameters.

### Sensitivity functions

To describe and obtain a non-parametrical description of the human balance control, the sensitivity function of the output of the human balance control (e.g. body sway, ankle torque, muscle activation) to the perturbation was obtained by estimating Frequency Response Functions (FRFs). Therefore, the perturbation, ankle torque, body sway, and muscle activation were transformed to the frequency domain. The PSDs and CSDs were computed to calculate the FRFs [25]. Only the excited frequencies were analysed. The FRFs were estimated using the indirect approach [25]:

2 $$ {}^{SS}{S}_x(f)={\varPhi}_{SS,x}(f)\cdotp {\left[{\varPhi}_{SS, SS}(f)\right]}^{-1} $$

In which Φ_*SS,x*_ represents the CSD of the SS rotation and *x*, which represents the ankle torque (T), body sway (BS) or muscle activation (MA), and Φ_*SS,SS*_ the PSD of the SS rotation. The FRF magnitude and the FRF phase represent the amplitude ratio and the relative delay, respectively, between the SS rotation and the ankle torque, body sway or muscle activation.

The individual FRFs of the experimental data were averaged across 20 participants resulting in one FRF per condition. The mean FRFs were used for further analysis.

### Parameter estimation

**Fig. 3:**
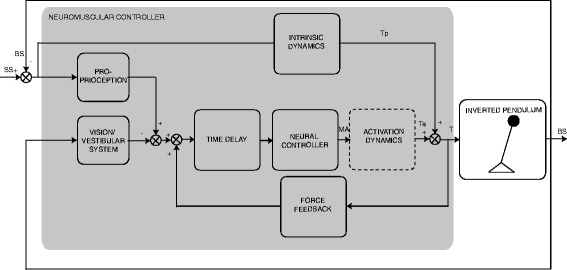
Model of the human balance control. The human body is modelled by an inverted pendulum controlled by the neuromuscular controller, consisting of a passive and active part. The passive part consists of the intrinsic dynamics representing the intrinsic properties of the muscles and produces a passive torque (T_p_). The active part consists of the neural controller with a time delay and activation dynamics producing an active torque (T_a_). The neural controller receives feedback from the sensory systems by force feedback (i.e. Golgi tendon organs) and position, velocity and acceleration feedback (i.e. proprioception, vision and vestibular system). The activation dynamics are added to the model simulation and in two of the three models used for parameter estimation

To give physiological meaning to the sensitivity functions, a human balance control model (Fig. 3) was used to describe the sensitivity functions. The balance control model was based on a previously described balance control model [13, 17] and consisted of a single inverted pendulum controlled by a neuromuscular controller using position and velocity feedback of the sensory systems, consisting of a time delay and neural controller. Dependent on the model that was used for fitting, the activation dynamics and the acceleration feedback were added to the model. Equation 3 shows the theoretical description of the sensitivity functions, i.e. the transfer functions.

3 $$ {\displaystyle \begin{array}{l}{{}^{SS}S}_{BS}\left(f,p\right)=\frac{BS(f)}{SS(f)}=\frac{P\cdotp BD+W\cdotp NC\cdotp ACT\cdotp BD}{1- FF\cdotp NC\cdotp ACT+P\cdotp BD+ NC\cdotp ACT\cdotp BD}\\ {}{{}^{SS}S}_T\left(f,p\right)=\frac{T(f)}{SS(f)}=\frac{P+W\cdotp NC\cdotp ACT}{1- FF\cdotp NC\cdotp ACT+P\cdotp BD+ NC\cdotp ACT\cdotp BD}\\ {}{{}^{SS}S}_{MA}\left(f,p\right)=\frac{MA(f)}{SS(f)}=\frac{W\cdotp NC+P\cdotp FF\cdotp NC-\left(1-W\right)\cdotp P\cdotp BD\cdotp NC}{1- FF\cdotp NC\cdotp ACT+P\cdotp BD+ NC\cdotp ACT\cdotp BD}\end{array}} $$

This model consists of several parameters describing the behaviour of the system. The parameters describing the sensitivity functions were estimated using the mathematical transfer functions of the balance control model (see Appendix). In this case, we are searching for model parameters such that the behaviour of the model matches with the experimentally measured behaviour.

Table 1 summarizes which model parameters were estimated and fixed during fitting. The mass and length were measured and used to calculate the CoM height defined as the length between the ankle axis and the CoM. The moment of inertia was calculated using the mass and length according to Winter (1990) [36]. The mass, CoM height and moment of inertia were used as fixed parameters. The parameters of the force feedback were fixed at a constant value to reduce interaction with the other estimated parameters.

**Table 1:** Overview of the estimated and fixed model parameters

Parameter	Estimated	Fixed
Moment of inertia		+
Body mass		+
Center of Mass height		+
Intrinsic stiffness	+	
Intrinsic damping	+	
Activation dynamics: Eigenfrequency	(+)	
Activation dynamics: Relative damping	(+)	
Time delay	+	
Proprioceptive weight	+	
Reflexive stiffness	+	
Reflexive damping	+	
Acceleration feedback	(+)	
Force feedback gain		+
Force feedback time constant		+

The model was fitted on the mean experimental FRFs (averaged across 20 participants) using a nonlinear least-square fit (Matlab function: lsqnonlin) by minimizing the sum squared error (E):

4 $$ {\displaystyle \begin{array}{l}\varepsilon \left(f,p\right)=\sqrt{\frac{\gamma_{SS,x}^2(f)}{f}}\cdotp \left|\log \left(\frac{S_{\mathrm{exp}}(f)}{S_{est}\left(f,p\right)}\right)\right|\\ {}E=\frac{1}{N}\varepsilon {\left(f,p\right)}^T\varepsilon \left(f,p\right)\end{array}} $$

In which γ_*SS,x*_^*2*^ represents the coherence between SS rotation and ankle torque, body sway or muscle activation, S_exp_(*f*) the experimental or simulated sensitivity function and S_est_(*f,p*) the estimated sensitivity function based on the estimated model parameters and *N* the number of estimated data points.

The coherence varies between 0 and 1, in which a coherence close to one reflects a good signal to noise ratio. By fitting the model on the experimental data, the gain of the EMG, the activation dynamics and the intrinsic dynamics were kept constant over conditions, i.e. they were condition-independent. All other parameters, i.e. reflexive dynamics, time delay and proprioceptive weight, varied over conditions and were therefore condition-dependent.

### Reliability

To evaluate the goodness of the model fits, first the Goodness of Fit (GOF) in the frequency domain was calculated for each sensitivity function using eq. 5.

5 $$ GOF=\left[1-\frac{\sum_{k=1}^N{\left|{S}_{est}\left({f}_k,p\right)-{S}_{\mathrm{exp}}\left({f}_k\right)\right|}^2}{\sum_{k=1}^N{\left|{S}_{est}\left({f}_k,p\right)\right|}^2}\right]\times 100\% $$

In which *S*_est_ *(f*_*k*_ *,p)* represents the estimated sensitivity function per frequency and parameter set and *S*_exp_ *(f*_*k*_ *)* the experimental sensitivity function per frequency.

Second, the Akaike information criterion (AIC) was calculated using eq. 6, which represents the relative quality of the different models and therefore the consequence of adding data and parameters to the parameter estimation. In other words, it shows whether adding parameters or data points to the analysis has additional value by increasing the quality of the model. The model does not necessarily explain more variance of the experimental data. A lower AIC indicates a higher quality of the model.

6 $$ AIC=\log (E)+\frac{2d}{N} $$

In which E is the summed squared error, N the number of estimated data points (i.e. the number of frequency points) and d the number of estimated parameters.

Third, the standard error of the mean (SEM) was calculated per parameter to describe the reliability of the parameters. A low SEM means a more precise estimation and therefore a low minimal detectable change. This indicates that the parameters are more sensitive for detecting differences between groups or changes within groups. The SEM was calculated using the diagonal of the estimated covariance matrix $\widehat{P}$ [37]:

7 $$ \widehat{P}=E{\left({J}^TJ\right)}^{-1} $$

In which J is the Jacobian (matrix of partial derivatives of the prediction error (ɛ, equation 4) to each parameter) and E the summed squared error. A high partial derivative corresponds with a low SEM; a change in the estimation results in a high change of the prediction error, which allows a more precise estimation.

The mean SEM (mSEM) was calculated by averaging the relative SEM values of the parameters, in which the relative SEM is obtained by dividing the SEM by the estimated value. To investigate the effect of adding the muscle activation and the acceleration feedback to the model on mSEM, mSEM was calculated for the activation dynamics parameters (mSEM ACT; relative damping and eigenfrequency), acceleration feedback parameter (mSEM K_a_) and for the other parameters (mSEM basic; intrinsic dynamics, reflexive dynamics, time delay and proprioceptive weight) separately.

### Model validation

A model validation study was performed to investigate the accuracy of the estimated parameters for all combinations of sensitivity functions and models by applying the method on simulated data using estimated model parameters obtained in the experiments. Therefore, one dataset was simulated using the same balance control model as used for the parameter estimation including the activation dynamics and acceleration feedback, which was implemented in Matlab (The Mathworks, Natick, USA) (Fig. 3 and Appendix). Pink noise was added according to Van der Kooij and Peterka [38] to mimic sensory and motor noise and simulated with Simulink. The parameters were set to specific values found in the human experiment of the condition with 2 degrees peak-to-peak perturbation amplitude and eyes open.

The model was perturbed with a sensory perturbation of the proprioceptive information by a continuous support surface rotation of 2 degrees peak-to-peak amplitude as described in detail in ‘Perturbation signal’. The simulation time was 2 min in which the perturbation signal was 6 times repeated. Time series of the support surface rotation, ankle torque, body sway and muscle activation were sampled at 1000 Hz and used for further analysis as explained in ‘Data analysis’ resulting in sensitivity functions and estimated model parameters.

### Accuracy and reliability

A comparison was made between the estimated values and the values used in the simulation represented by the relative difference per parameter. These differences were averaged representing the mean relative difference (mDIFF) indicating the accuracy. mDIFF was calculated for the activation dynamics parameters (mDIFF ACT), the acceleration feedback parameter (mDIFF K_a_) and for the other parameters (mDIFF basic), separately.

To assess the reliability, the same measures were calculated as for the experimental data (i.e. the GOF, AIC, mSEM basic, mSEM ACT and mSEM K_a_).

## Results

A human experiment was performed to show the effect on the estimated parameters by adding muscle activation and therefore activation dynamics and acceleration feedback to CLSIT. Next, a model validation study was performed to investigate the accuracy. Below, the results of both studies are described.

### Human experiment

**Fig. 4:**
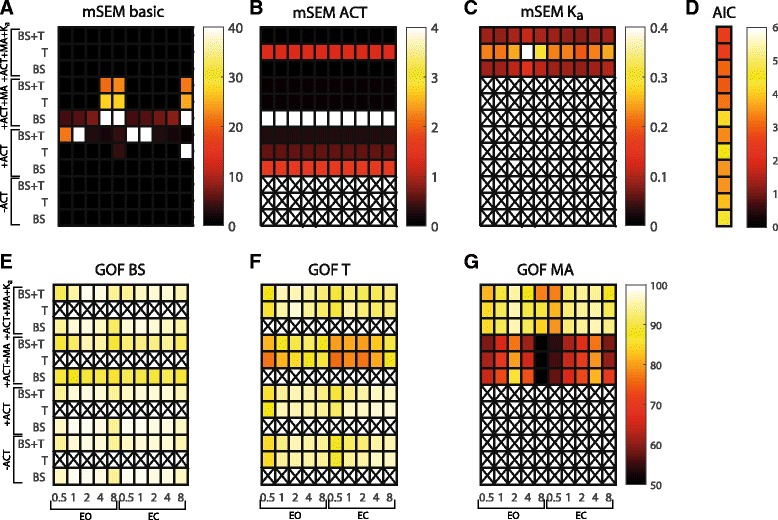
Overview of the mean standard error of the mean (SEM) of the basic parameters (**a**), of the activation dynamics parameters (**b**) and of the acceleration feedback (**c**), the Akaike information criterium (AIC) (**d**) and the goodness of fit (GOF) (**e**, **f** and **g**) for each condition and for each combination of sensitivity functions and fitted models. EO: eyes open, EC: eyes closed, BS: body sway, T: ankle torque, MA: muscle activation, ACT: activation dynamics, K_a_: acceleration feedback, X: not applicable

Figure 4 gives an overview of the goodness of parameter estimation on experimental data for all 10 conditions (0.5, 1, 2, 4 and 8 degrees peak-to-peak amplitude with eyes open and eyes closed) in terms of mSEM, AIC and GOF. Adding the activation dynamics to the fitted model results in a higher mSEM of the basic parameters compared with a model without activation dynamics, especially when the torque sensitivity function is used in combination or not with the body sway sensitivity function. Adding also the sensitivity function of the muscle activation to the fitting procedure results in a lower mSEM of the basic parameters, except when only the body sway sensitivity function is used. Adding the acceleration feedback in the fitted model resulted in a low mSEM of the basic parameters, which is comparable with the mSEM without activation dynamics. The mSEM of the activation dynamics parameters is only low for the torque sensitivity function or combined with the body sway sensitivity function. In case also the acceleration feedback is added, the mSEM of the activation dynamics is only low when the body sway sensitivity function or combined with the torque sensitivity function is used. The relative SEM of the acceleration feedback itself was also low in case the body sway sensitivity function was used or in combination with the torque sensitivity function (< 0.14).

**Fig. 5:**
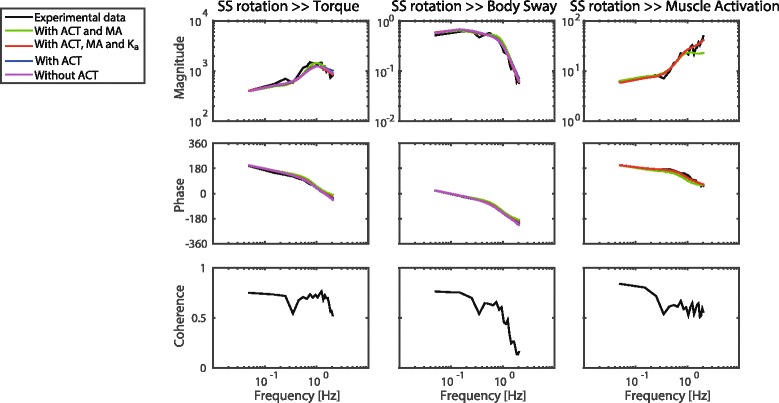
Frequency Response Functions of the experimental data with the fitted models of one specific condition (2 degrees peak-to-peak amplitude with eyes closed). Results are only shown of the model fits in which both the sensitivity function of the SS rotation to torque and of the SS rotation to body sway were used. SS: support surface, MA: muscle activation, ACT: activation dynamics, K_a_: acceleration feedback

Adding the activation dynamics did not affect the GOF compared with parameter estimation without activation dynamics. Adding the sensitivity function of the muscle activation affected the GOF of the sensitivity function of the body sway and of the ankle torque. Also a low GOF of the muscle activation was found. Adding the acceleration feedback results again in a high GOF for all sensitivity functions. These results are also shown in Fig. 5; the fit on the sensitivity functions are comparable for all combinations of sensitivity functions and models, with the best fit in case the acceleration feedback is added to the model.

Adding the activation dynamics did not influence the AIC of the fits substantially. Adding also the muscle activation resulted in a lower AIC, which decreased even more with adding the acceleration feedback.

**Fig. 6:**
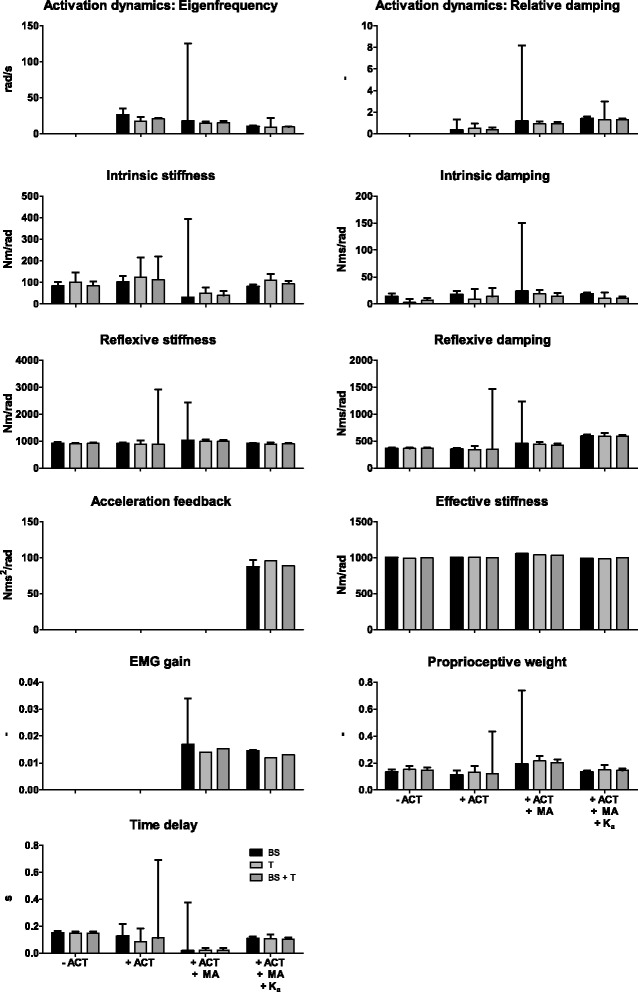
Overview of estimated parameters from the experimental data for each combination of sensitivity functions by adding activation dynamics, muscle activation and acceleration feedback to closed loop system identification. Parameter values are given with standard error of the mean (SEM) of one condition (2 degrees peak-to-peak amplitude with eyes open (EO)). BS: body sway, T: ankle torque, MA: muscle activation, ACT: activation dynamics, K_a_: acceleration feedback

Figure 6 shows the results of the parameter estimation of one condition (2 degrees peak-to-peak amplitude with eyes open) with adding the activation dynamics, the muscle activation and the acceleration feedback to CLSIT. Additional files 1 and 2 show the results of all conditions. Adding the activation dynamics in the fitted model results in comparable estimates as the estimates without the activation dynamics. However, the time delay, the proprioceptive weight and intrinsic stiffness show a high SEM with the addition of activation dynamics.

Adding the muscle activation results in a lower estimate of the intrinsic dynamics and time delay and a higher estimate of the proprioceptive weight. Furthermore, a high SEM of the activation dynamics was found. The SEM of the time delay and proprioceptive weight was lower compared with the situation without use of the muscle activation sensitivity function, except when the body sway sensitivity function was combined with the muscle activation sensitivity function. Adding the acceleration feedback shows low SEMs for all estimated parameters, except when only the torque sensitivity function was used in combination with the muscle activation sensitivity function. In contrast with the fitted model without acceleration feedback, higher values are found for the time delay and intrinsic dynamics.

### Model validation

Table 2 shows the goodness of the parameter estimation on the simulated data with each combination of sensitivity functions and the fitted model including the activation dynamics and acceleration feedback or not. A model fit without activation dynamics and without the use of the muscle activation shows a high mDIFF (22.2–22.4%). Adding the activation dynamics to the fitted model increased the mean difference of the basic parameters (23.3–28.0%) and resulted in a high mDIFF of the activation dynamics parameters (i.e. the relative damping and eigenfrequency) (47.6–59.4%).

**Table 2:** Overview of mean relative differences (mDIFF), mean standard error of the mean (mSEM), goodness of fit (GOF) and Akaike information criterium (AIC) for each combination of sensitivity functions and models fitted on simulated data

	mDIFF (%)			mSEM			GOF (%)			AIC
	Basic^a^	ACT^b^	K_a_	Basic^a^	ACT^b^	K_a_	BS	T	MA	
Fitted model without activation dynamics										
BS	22.4	–	–	0.10	–	–	99.5	–	–	−1.34
T	22.2	–	–	0.11	–	–	–	99.4	–	−1.36
BS + T	22.2	–	–	0.08	–	–	99.5	99.4	–	−0.94
Fitted model with activation dynamics										
BS	28.0	47.6	–	1.76	0.44	–	99.7	–	–	−1.43
T	27.6	47.9	–	1.66	0.42	–	–	99.5	–	−1.45
BS + T	23.3	59.4	–	0.46	0.26	–	99.7	99.5	–	−1.11
BS + MA	47.4	30.9	–	1.94e^12^	0.27	–	93.5	–	85.6	1.88
T + MA	47.5	30.9	–	2.19e^12^	0.28	–	–	90.9	85.6	1.88
BS + T + MA	48.2	31.0	–	2.01e^12^	0.22	–	96.2	95.2	81.6	2.02
Fitted model with activation dynamics and acceleration feedback										
BS + MA	15.0	0.8	15.4	0.08	0.09	0.07	99.5	–	98.7	−0.41
T + MA	15.1	0.8	15.4	0.08	0.09	0.07	–	99.3	98.7	−0.43
BS + T + MA	13.1	0.7	13.9	0.07	0.08	0.07	99.6	99.5	98.5	−0.25

*BS* body sway, *T* ankle torque, *MA* muscle activation, *ACT* activation dynamics, *K*_*a*_ acceleration feedback

^a^Basic parameters consist of intrinsic dynamics, reflexive dynamics, time delay and proprioceptive weight

^b^Activation dynamics parameters consist of the relative damping and eigenfrequency

Adding also the muscle activation to the fitting procedure resulted in a higher mDIFF of the basic parameters (47.4–48.2%) and a smaller mDIFF of the activation dynamics parameters (30.9–31.0%). When also the acceleration feedback was added to the fitted model, a decrease of the mDIFF of both the basic parameters (13.1–15.1%) and the activation dynamics parameters (0.7–0.8%) was found. Also, the mDIFF of the acceleration feedback parameter was small (13.9–15.4%).

Adding the activation dynamics to the fitted model resulted in a higher mSEM of the basic parameters (0.46–1.76) compared with a fitted model without the activation dynamics (0.08–0.11). Adding also the muscle activation to the fitting procedure resulted in an even higher mSEM of the basic parameters (1.94 e^12^–2.19 e^12^). When also the acceleration feedback was added to the fitted model, again a low mSEM (0.07–0.08) was found, which is comparable with the low mSEM as found before, without muscle activation and activation dynamics. The mSEM of the activation dynamics decreased with adding the muscle activation from 0.26–0.44 to 0.22–0.28 and decreased even more with adding the acceleration feedback to 0.08–0.09. The mSEM of the acceleration feedback parameter was low (0.07).

Adding the activation dynamics in the fitted model did not affect the GOF of the sensitivity functions of the body sway and the ankle torque substantially. However, adding the muscle activation to the fitting procedure resulted in a decrease of the GOF. Also adding the acceleration feedback to the fitted model resulted again in a high GOF of all sensitivity functions.

The AIC of the parameter estimation increased with adding the muscle activation, i.e. adding data points. The AIC decreased again with adding also the acceleration feedback to the fitted model, i.e. adding parameters.

**Fig. 7:**
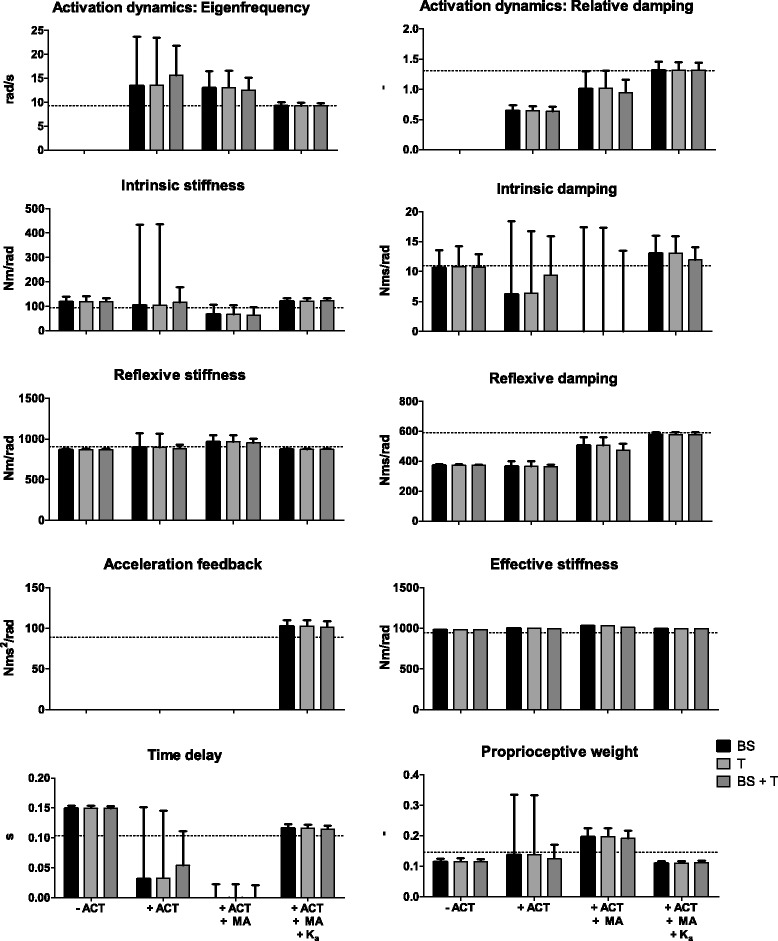
Overview of estimated parameters from the simulated data and parameter values used in simulation for each combination of sensitivity functions and fitted models. Parameter values are given with standard error of the mean (SEM). The dotted line indicates the parameter values used in the model simulation. BS: body sway, T: ankle torque, MA: muscle activation, ACT: activation dynamics, K_a_: acceleration feedback

Figure 7 shows the comparison between the estimated parameters and the parameters used in the simulation with the corresponding SEM. Without activation dynamics in the fitted model and muscle activation in the fitting procedure, the intrinsic stiffness and time delay are estimated higher compared to the original value. The reflexive dynamics (i.e. the reflexive stiffness and reflexive damping) and the proprioceptive weight are estimated lower, while the intrinsic damping is comparable with the original parameter. Adding the activation dynamics to the fitted model resulted in even more differences between the estimated and original values, i.e. the time delay, intrinsic damping and activation dynamics parameters were not comparable. Furthermore, the intrinsic dynamics, proprioceptive weight and time delay show a high SEM. Adding also the muscle activation to the fitting procedure results in less difference between the estimates of the activation dynamics and reflexive damping. However, the intrinsic damping and time delay were much lower than the original value. Furthermore, a lower SEM was found for the proprioceptive weight, intrinsic stiffness and time delay. Adding the acceleration feedback to the fitted model resulted in more comparable estimates and also a low SEM for all parameters. The effective stiffness (i.e. the sum of the intrinsic and reflexive stiffness) is comparable with the original effective stiffness as used in the simulation in all situations.

## Discussion

In this study we investigated the additional value of adding EMG of the lower legs (representing the muscle activation) and the activation dynamics and acceleration feedback to CLSIT and parameter estimation to assess the contribution of the underlying systems involved in standing balance. With experimental data we showed that both the activation dynamics and acceleration feedback must be added to the fitted model in combination with the muscle activation to the parameter estimation to obtain a reliable estimate of the parameters describing the underlying systems in standing balance. A model validation study confirmed these findings and showed that this resulted in more accurate estimated parameters describing the underlying systems in standing balance.

### Additional value of muscle activation, activation dynamics and acceleration feedback

Adding the activation dynamics to the fitted model is only of additional value, when this addition is accompanied by addition of the muscle activation to the parameter estimation and the acceleration feedback to the fitted model. Adding only the activation dynamics result in a higher mSEM due to the less reliable estimation of the intrinsic stiffness, time delay and proprioceptive weight. This indicates an over parameterization of the model, as the goodness of fit was not influenced. When also the muscle activation was added in the parameter estimation, the mSEM decreases again, which indicates a more reliable estimation of the intrinsic stiffness, time delay and proprioceptive weight. However, a low and physiologically unexplainable value of the time delay was found. As also the GOF shows low values for the sensitivity function of the ankle torque and the muscle activation, which indicates that the model does not describe the experimental data well, we added an acceleration feedback to the fitted model. Previous studies already showed that the muscle activation must be explained by acceleration feedback, next to position and velocity feedback [31, 32]. Adding this parameter resulted in a higher GOF, a more realistic time delay and a lower AIC, which indicates that the quality of the model increased.

Adding the activation dynamics and acceleration feedback did not influence the estimates of the proprioceptive weight, which indicates that the proprioceptive weight can reliably be identified with addition of these parameters. The estimated proprioceptive weight is comparable with previous studies [13, 17].

### Distinction between neural time delay and electromechanical delay

Including the activation dynamics to the fitted model used for parameter estimation, makes it possible to separate the neural time delay and the electromechanical delay due to the activation dynamics. Adding only the activation dynamics to the fitted model resulted in less reliable estimated time delay indicated by a high SEM. Adding also the muscle activation to the fitting procedure resulted in more reliable estimated parameters but an unrealistically low time delay (<0.001 s) compared with previous studies [15–17]. Previous studies showed that the effective time delay is around 200 ms [17], of which the time delay of the muscle activation is around 12 ms [39]. As in the current study both the neural time delay and electromechanical time delay were separated by including the activation dynamics, we expected to find a neural time delay around 190 ms. By adding the acceleration feedback in the fitted model, more physiologically realistic and reliable values of the time delay were found.

The activation dynamics estimated with addition of the acceleration feedback are comparable with previous studies in which the activation dynamics is modelled as a second order system [22, 32, 40].

### Distinction between intrinsic and reflexive contributions

It was expected that adding the activation dynamics to the fitted model and the muscle activation to the parameter estimation would result in more accurate and reliable estimates of the intrinsic and reflexive dynamics. The results of the human experiment indeed shows reliable estimates of the intrinsic and reflexive dynamics in all situations, except in case only the activation dynamics are added. However, the model validation study shows that these parameters are only accurately estimated when adding the activation dynamics and acceleration feedback to the fitted model in combination with the muscle activation.

The sum of the estimated reflexive and intrinsic stiffness (i.e. the effective stiffness, Fig. 6) was comparable for all models and combinations of sensitivity functions. This indicates that by using these models and sensitivity functions it is possible to estimate the sum of the reflexive and intrinsic stiffness. However, for separating these parameters, muscle activation needs to be added to the parameter estimation, and acceleration feedback and activation dynamics should be added to the fitted model.

A low value was found for the estimation of the intrinsic stiffness and damping of all combinations of sensitivity functions without activation dynamics. Including the activation dynamics into the fitted model resulted in physiologically more realistic intrinsic dynamics. Previous studies estimating the balance behaviour, which did not add the muscle activation, showed that it is difficult to separate the intrinsic and reflexive dynamics using parameter estimation. Peterka (2002) showed the results of a model with and without the intrinsic properties. He indicated that including the intrinsic properties did not always result in good fits [17]. Furthermore, Pasma et al. (2015) and Engelhart et al. (2016) did not add the intrinsic properties in their models, due to unreliable estimates of the intrinsic properties [13, 14]. Also Kiemel et al. (2008), who did add the muscle activation, indicated that the intrinsic damping might to be too small to detect [22]. However, adding the activation dynamics in combination with the muscle activation and the acceleration feedback results in more accurate and physiologically meaningful parameters.

### Effect of increasing perturbation amplitude and closing the eyes

Previous studies showed that with increasing perturbation amplitude and closing the eyes sensory reweighting, which is an important aspect of standing balance, can be investigated. By sensory reweighting the contribution of sensory systems changes based on the accuracy of the information that these systems receive. Additional files 1 and 2 show the estimated parameters, obtained with all combinations of sensitivity functions and models, of all conditions with increasing perturbation amplitude performed with eyes open and eyes closed.

The results show that the proprioceptive weight decreased with increasing perturbation amplitude (i.e. sensory reweighting), which is in accordance with previous studies [13, 17]. This phenomenon is clearly visible and is not influenced by the addition of the activation dynamics and acceleration feedback to the fitted model and the muscle activation to the parameter estimation. This result suggests that with all used models and combinations of sensitivity functions, sensory reweighting can be quantified.

Also the time delay decreases with increasing perturbation amplitude, which is in accordance with previous studies [17]. An explanation for this might be the difference in time delay between each sensory pathway. With increasing perturbation amplitude, proprioception will contribute less while the other sensory systems contribute more. This will result in a change of the transporting time of the sensory information and therefore the time delay [17].

The intrinsic dynamics were kept constant over conditions, resulting in condition-independent parameters. Keeping these parameters constant resulted in smaller number of estimated parameters. The AIC showed that the quality of the fitted model remains the same with fewer parameters (data not shown).

### Experimental set up

In this study the parameter estimation was performed with several combinations of sensitivity functions. The results showed that including only the ankle torque or body sway or a combination of these influences the reliability of the estimated parameters. In case the activation dynamics is combined with the muscle activation, the use of only the body sway shows an unreliable estimate of all parameters, while adding the acceleration feedback makes the use of only the torque less reliable for estimating all parameters. These results indicate that the reliability depends on the fitted model and the included time series used in CLSIT and parameter estimation.

### Model validation

The aforementioned results are confirmed by the model validation study. The results of the model validation study show that both the neural time delay and activation dynamics can be estimated reliably and accurately in case both the activation dynamics and acceleration feedback are added to the fitted model in combination with the muscle activation. This also allows for a reliable and accurate distinction between the intrinsic and reflexive dynamics.

The results of the model validation study did not confirm the observation that it depends on the fitted model which sensitivity functions must be added to get reliable estimated parameters. The parameters are estimated with the same reliability and accuracy with all combinations of sensitivity functions.

### Recommendations

We recommend to add activation dynamics, acceleration feedback and muscle activation to the fitting procedure to accurately and reliably separate the time delay due to the activation dynamics and the neural time delay, and the intrinsic and reflexive dynamics. We therefore expect that using such a model in clinical populations could help to distinguish the different underlying causes of impaired balance found with age and disease and therefore to specifically diagnose impaired balance and develop and apply targeted interventions to improve standing balance [13, 41].

It is recommended to combine EMG measurements with a fitted model including acceleration feedback in the neural controller. This is in contrast with previous studies using system identification and parameter estimation. Welch et al. (2008) showed that the muscle activation during stance must be explained by a position, velocity and acceleration feedback gain. Ignoring the acceleration feedback gain results in a low and a not physiologically realistic time delay [31], which is in agreement with our results.

When no EMG is available it is recommended to exclude the activation dynamics and acceleration feedback from the parameter estimation or give them fixed values obtained from literature, to minimize the effect on the accuracy and reliability of the estimation of the activation dynamics, time delay and proprioceptive weight. On the other hand, it is not recommended to use only muscle activation in CLSIT as this results in unreliable and less accurate estimations of all parameters (data not shown).

The results show low values of the intrinsic properties, which is against expectation [42]. This might be due to the used perturbation signal. The intrinsic dynamics are modelled as a stiffness and damping without a time delay and mainly affects the low frequencies. As the low frequencies are underrepresented, a perturbation signal containing more low frequencies might result in a better estimation of the intrinsic dynamics. Therefore, we recommend to investigate the effect of the perturbation signal in general on the parameter estimation.

We recommend to always consider carefully which model and data you will use to describe human balance behaviour, as the results show that it depends on the fitted model whether the muscle activation must be combined with the body sway, ankle torque or both to obtain the most reliable estimated parameters. To get the most accurate and reliable estimated parameters, it is recommended to combine muscle activation with at least body sway measurements. This means that a motion capture system or another method to measure the body sway must be combined with EMG and ideally with force plates.

## Conclusions

In conclusion, adding the activation dynamics and acceleration feedback to the fitted model and the muscle activation to CLSIT improves the accuracy and reliability of the estimated parameters describing the underlying systems in standing balance. This shows that it is possible to separate the muscle activation dynamics from the neural time delay and the intrinsic dynamics from the reflexive dynamics, which gives more insight in the contribution of the underlying systems in standing balance. More detailed information about the underlying systems and therefore the underlying changes with age and disease gives the opportunity to diagnose impaired balance more specifically and improve standing balance with targeted interventions. Therefore, it is recommended to measure EMG in combination with body sway (with or without ankle torque) and add activation dynamics and acceleration feedback to the fitted model used for parameter estimation to assess the underlying systems involved in standing balance and to improve diagnosis of impaired balance.

### Additional files

Additional file 1: Figure with estimated parameters from the experimental data of all conditions for each combination of sensitivity functions by adding activation dynamics in the fitted model. Parameter values are given with standard error of the mean (SEM) of 10 conditions (0.5, 1, 2, 4 and 8 degrees peak-to-peak amplitude with eyes open (EO) and eyes closed (EC)). BS: body sway, T: ankle torque, ACT: activation dynamics. (PDF 74 kb)

Additional file 2: Figure with estimated parameters from the experimental data of all conditions for each combination of sensitivity functions by adding muscle activation and acceleration feedback in the fitted model. Parameter values are given with standard error of the mean (SEM) of 10 conditions (0.5, 1, 2, 4 and 8 degrees peak-to-peak amplitude with eyes open (EO) and eyes closed (EC)). BS: body sway, T: ankle torque, MA: muscle activation, ACT: activation dynamics, K_a_: acceleration feedback. (PDF 66 kb)

## Appendix

The balance control model used in this study is adapted from previous publications [13, 17]. In this model the human body is modelled as an inverted pendulum (eq. 8).

8 $$ BD=\frac{1}{Js^2- mgh} $$

In which m represents the body mass in kg above the feet, h represents the Center of Mass height in m and J the moment of inertia in kgm^2^.

This inverted pendulum is controlled by the neuromuscular controller consisting of an active and passive part. The passive part represents the intrinsic dynamics of the muscles and the tendon modelled by an intrinsic stiffness (K) in Nm/rad and intrinsic damping (D) in Nms/rad (eq. 9).

9 $$ P=K+ Ds $$

The active part is the neural controller and represents the reflexive contribution of the muscles modelled by a controller, consisting of a reflexive stiffness (K_p_) in Nm/rad, reflexive damping (K_d_) in Nms/rad and acceleration feedback (K_a_) in Nms^2^/rad, with a time delay (τ) in s (eq. 10).

10 $$ NC=\left({K}_p+{K}_ds+{K}_a{s}^2\right)\cdot {e}^{-\tau s} $$

The neural controller receives feedback information about the body position and velocity and forces from the sensory systems. The Golgi tendon organs provide a force feedback, which is represented by a low pass filter with a time constant (τ_f_) in s and a unit less gain (K_f_) (eq. 11).

11 $$ FF=\frac{K_f}{\tau_fs+1} $$

Position and velocity feedback is provided by the proprioception, vision and vestibular system. The sensory reweighting theory says that all information is weighted by a weight W in which the sum of the sensory weights equals one. All feedback is used by the neural controller to generate a motor command and sent this to the muscles. The muscles will activate and generate a corrective torque. The muscle activation dynamics is modelled by a second order system consisting of the eigenfrequency (f_0_) in Hz and a unit less relative damping (β) (eq. 12).

12 $$ ACT=\frac{\omega^2}{s^2+2\beta \omega s+{\omega}^2},{\omega}^2=2\pi {f}_0 $$

ω indicates the natural frequency, i.e. the frequency at which the system starts to oscillate in case the relative damping β will be zero. Due to the activation of the muscles, the body position will change and information about the new situation is sensed by the sensory systems and sent to the neural controller. So, the loop starts over and we have to do with a closed loop system.

To represent the sensitivity of the body sway (BS), ankle torque (T) and muscle activation (MA) to the support surface (SS) rotation, the sensitivity functions could be calculated according to eq. 13.

13 $$ {\displaystyle \begin{array}{l}{{}^{SS}S}_{BS}(s)=\frac{BS(s)}{SS(s)}=\frac{P\cdotp BD+W\cdotp NC\cdotp ACT\cdotp BD}{1- FF\cdotp NC\cdotp ACT+P\cdotp BD+ NC\cdotp ACT\cdotp BD}\\ {}{{}^{SS}S}_T(s)=\frac{T(s)}{SS(s)}=\frac{P+W\cdotp NC\cdotp ACT}{1- FF\cdotp NC\cdotp ACT+P\cdotp BD+ NC\cdotp ACT\cdotp BD}\\ {}{{}^{SS}S}_{MA}(s)=\frac{MA(s)}{SS(s)}=\frac{W\cdotp NC+P\cdotp FF\cdotp NC-\left(1-W\right)\cdotp P\cdotp BD\cdotp NC}{1- FF\cdotp NC\cdotp ACT+P\cdotp BD+ NC\cdotp ACT\cdotp BD}\end{array}} $$

As measured EMG signals are in Volt (V) and does not represent the muscle activation, an EMG gain (K_EMG_) is added to the sensitivity functions and estimated to translate the measured Volts to the muscle activation (eq. 14).

14 $$ {{}^{SS}S}_{MA}(s)=\frac{MA(s)}{SS(s)}={K}_{EMG}\cdotp \frac{W\cdotp NC+P\cdotp FF\cdotp NC-\left(1-W\right)\cdotp P\cdotp BD\cdotp NC}{1- FF\cdotp NC\cdotp ACT+P\cdotp BD+ NC\cdotp ACT\cdotp BD} $$

The above mathematical description of the balance behaviour is used to simulate human balance control and to describe the simulated or experimental data by searching for model parameters such that the behaviour of the model matches with the simulated or experimental measured behaviour. In the simulation, parameter values were used as found in the experimental data (Table 3).

**Table 3:** Overview of the model parameters with their values used in data simulation

Parameter	Symbol	Value
Moment of inertia	J	80.8 [kg m^2^]
Body mass	m	70.1 [kg]
Center of Mass height	h	0.99 [m]
Intrinsic stiffness	K	94.4 [Nm/rad]
Intrinsic damping	D	10.9 [Nms/rad]
Activation dynamics: Eigenfrequency	f_0_	1.48 [Hz]
Activation dynamics: Relative damping	β	1.30 [−]
Time delay	τ_d_	0.104 [s]
Proprioceptive weight	W	0.145 [−]
Reflexive stiffness	K_p_	908.2 [Nm/rad]
Reflexive damping	K_d_	591.7 [Nms/rad]
Acceleration feedback	K_a_	89.1 [Nms^2^/rad]
Force feedback gain	K_f_	0.0018 [rad/Nm]
Force feedback time constant	τ_f_	17.4 [s]

## Abbreviations

ACT: Activation dynamics
BAP: Bilateral Ankle Perturbator
BS: Body sway
CLSIT: Closed loop system identification
CoM: Center of Mass
CSD: Cross Spectral Density
DIFF: difference
EMG: Electromyography
FRF: Frequency Response Function
GOF: Goodness of Fit
MA: Muscle activation
PRTS: Pseudorandom ternary sequence
PSD: Power Spectral Density
SEM: Standard Error of the Mean
SS: Support surface
T: Torque
