# Supplementary material for: Assessment of the underlying systems involved in standing balance: the additional value of electromyography in system identification and parameter estimation
Source: J Neuroeng Rehabil. 2017 Sep 15;14:97. doi: 10.1186/s12984-017-0299-x (PMC5603100; doi:10.1186/s12984-017-0299-x)
Supplement: Supplementary file 2 — Figure with estimated parameters from the experimental data of all conditions for each combination of sensitivity functions by adding muscle activation and acceleration feedback in the fitted model. Parameter values are given with standard error of the mean (SEM) of 10 conditions (0.5, 1, 2, 4 and 8 degrees peak-to-peak amplitude with eyes open (EO) and eyes closed (EC)). BS: body sway, T: ankle torque, MA: muscle activation, ACT: activation dynamics, Ka: acceleration feedback. (PDF 66 kb) [file 12984_2017_299_MOESM2_ESM.pdf]

Additional file 2: Figure with estimated parameters from the experimental data of all conditions for each combination of sensitivity functions by adding muscle activation and acceleration feedback in the fitted model.

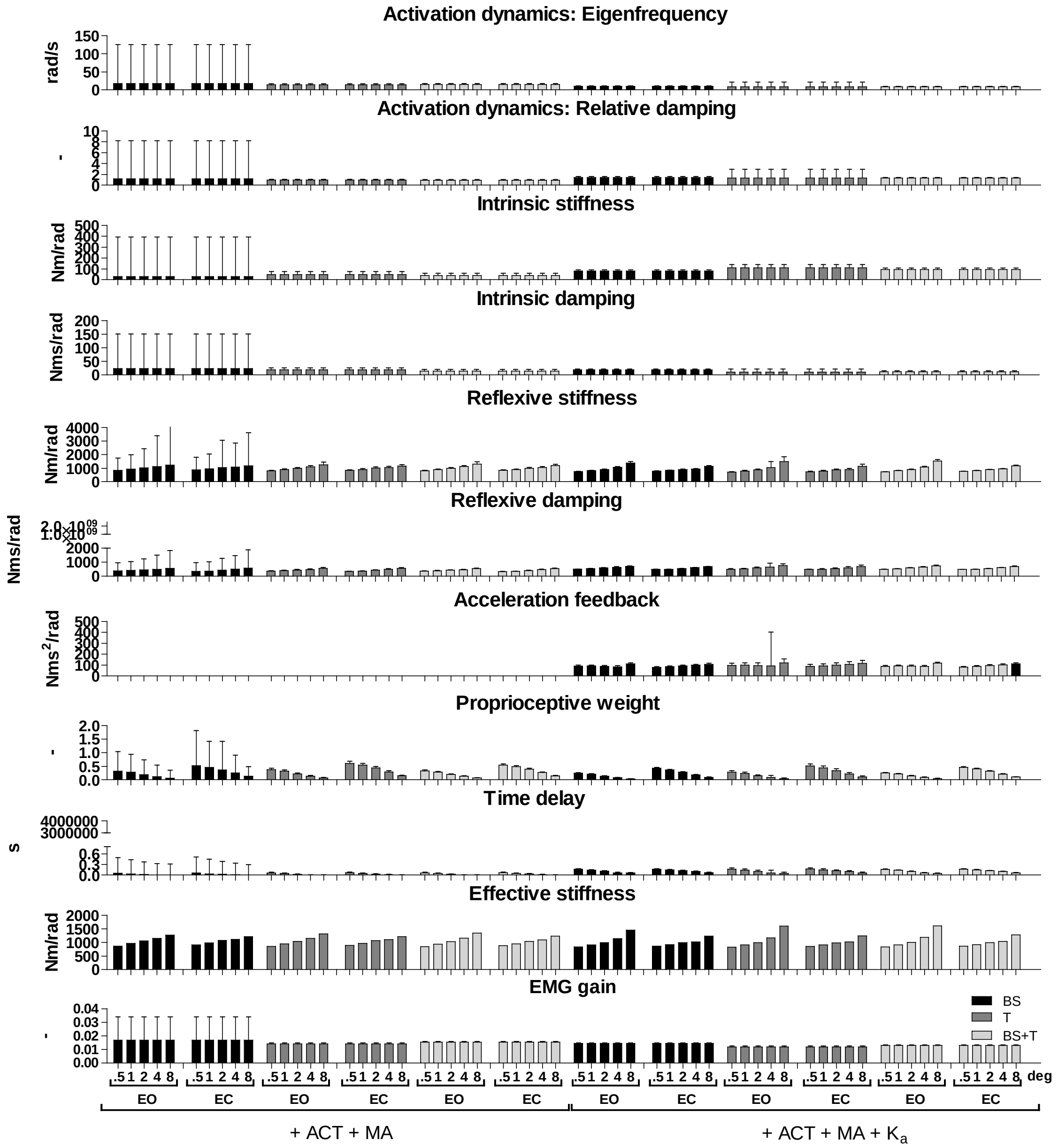

Parameter values are given with standard error of the mean (SEM) of 10 conditions (0.5, 1, 2, 4 and 8 degrees peak-to-peak amplitude with eyes open (EO) and eyes closed (EC)). BS: body sway, T: ankle torque, MA: muscle activation, ACT: activation dynamics, K_a: acceleration feedback.
